# Factors Associated With Fear of Cancer Recurrence in a Multiethnic Cohort of Patients With Breast Cancer

**DOI:** 10.1002/pon.70307

**Published:** 2025-10-16

**Authors:** Armaan Jamal, Fangyuan Zhao, Jincong Q. Freeman, Yijia Sun, Marcia M. Tan, Rita Nanda, Nan Chen, Olufunmilayo I. Olopade, Dezheng Huo

**Affiliations:** ^1^ Department of Public Health Sciences University of Chicago Chicago Illinois USA; ^2^ Cancer Prevention and Control Program UChicago Medicine Comprehensive Cancer Center Chicago Illinois USA; ^3^ Center for Health and the Social Sciences University of Chicago Chicago Illinois USA; ^4^ Section of Hematology & Oncology Department of Medicine University of Chicago Chicago Illinois USA; ^5^ Center for Innovation in Global Health University of Chicago Chicago Illinois USA

**Keywords:** breast cancer, cross‐sectional study, fear of cancer recurrence, psychosocial factors, survivorship

## Abstract

**Background:**

Fear of cancer recurrence (FCR) is prevalent among patients with breast cancer, yet those at high risk are not well characterized.

**Aims:**

This study aimed to identify the patient characteristics associated with FCR after diagnosis.

**Methods:**

Between July and September 2023, participants in the Chicago Multiethnic Epidemiologic Breast Cancer Cohort completed the 9‐item FCR‐Short Form Inventory. A score of 22 or higher indicated clinically significant FCR (csFCR). Logistic and linear regression estimated associations between different risk factors and csFCR and FCR scores, respectively. Missing data were addressed using multiple imputation.

**Results:**

Among 1390 stage I–III patients (mean age 63.1 years and 9.1 [mean] years since diagnosis), 262 (18.8%) reported csFCR. In adjusted models, csFCR was associated with having another cancer excluding non‐melanoma skin (adjusted odds ratio [aOR], 2.64; 95% CI, 1.66–4.21), greater levels of stress (aOR, 2.40 per 1‐SD increment; 95% CI, 1.98–2.91), having a prior recurrence (aOR, 2.26; 95% CI, 1.14–4.47), and having estrogen receptor‐positive tumors (aOR, 1.60; 95% CI, 1.06–2.40). Older age at diagnosis (aOR, 0.64 per 10‐year increment; 95% CI, 0.56–0.73) was associated with lower odds of csFCR. Similar associations were observed with continuous FCR scores, along with advanced stage at diagnosis associated with higher FCR scores (*p*‐trend = 0.001).

**Conclusions:**

Nearly 1 in 5 breast cancer patients reported csFCR. Key risk factors included younger age at diagnosis, history of recurrence or other malignancies, greater levels of stress, and estrogen receptor‐positive breast cancer, which can inform targeted interventions to support survivorship.

## 1 Introduction

Breast cancer remains one of the most prevalent cancers affecting women worldwide [1]. Although advances in early detection and treatment have significantly improved survival rates, the consequences of treatment often include persistent physical symptoms, functional impairments, and psychological distress [2, 3]. Fear of cancer recurrence (FCR) is defined as “fear, worry, or concern relating to the possibility that a primary cancer or subsequent malignancy will return or progress to other parts of the body” and is prevalent among patients with adult‐onset cancers [4]. Among patients with breast cancer in particular, FCR is one of the most frequently reported unmet needs, with significant implications for mental health, treatment adherence, and overall quality of life [5, 6].

FCR is influenced by a broad range of psychosocial and clinical factors, including degree of emotional or social support, persistent physical symptoms, and varied perceptions of illness [7]. Elevated FCR is also associated with excessive and potentially avoidable use of healthcare services [8]. Sequelae of a cancer diagnosis and treatment may contribute to heightened vulnerability to FCR, which in turn can adversely impact emotional well‐being and even long‐term clinical outcomes [5, 6]. FCR can significantly disrupt patients' daily lives, contributing to emotional distress, strained interpersonal relationships, family conflict, and diminished capacity to fulfill familial responsibilities, which negatively affect both patients and their families [9]. Clinically significant FCR (csFCR), defined as fear that leads to functional impairment or considerable distress, has been consistently associated with poor psychological outcomes such as anxiety, depression, reduced quality of life, and increased outpatient service use [10]. Risk factors for FCR include younger age, female sex, metastatic disease, persistent physical symptoms (e.g., fatigue, pain), and certain treatment exposures, particularly chemotherapy [7]. While several risk factors for FCR have been identified in other cancer populations, less is known about how these and other potential predictors manifest specifically among breast cancer survivors. Furthermore, most FCR studies in breast cancer have focused on predominantly non‐Hispanic White populations, limiting generalizability. Notably, prior research on long‐term survivors has been conducted primarily outside the United States (U.S.). Our study addresses these gaps by examining FCR in a racially and ethnically diverse U.S. breast cancer cohort.

The usage of poorly validated and brief tools has also limited efforts to reliably identify individuals experiencing csFCR and to understand its broader implications. Employing a validated and comprehensive measure of FCR, we addressed this gap by examining the correlates of both csFCR and FCR severity in a large, diverse cohort of individuals diagnosed with breast cancer. Guided by Lazarus and Folkman's transactional model of stress and coping, which conceptualizes health outcomes as shaped by the interplay between stressors, individuals' appraisal of those stressors, and available coping resources, we sought to identify both clinical and psychosocial predictors of FCR [11]. By identifying key predictors of FCR, we aim to deepen understanding of the psychological experiences of breast cancer patients and survivors, and inform the design of culturally sensitive, evidence‐based interventions to support survivorship care.

## 2 Materials & Methods

### 2.1 Design, Setting, and Participants

Data used in this study were drawn from the Chicago Multiethnic Epidemiologic Breast Cancer Cohort (ChiMEC). ChiMEC is a hospital‐based longitudinal study that consists of over 4700 patients with breast cancer who were prospectively identified and recruited at the time of diagnosis at the University of Chicago Medicine since 2008. Details on the ChiMEC cohort are described comprehensively elsewhere [12]. Between July and September 2023, eligible ChiMEC participants were surveyed via REDCap or postal mail and followed up with phone calls. The survey asked participants to respond to the 9‐item FCR‐Short Form Inventory (FCRI‐SF). Other information, such as chronic health conditions, BMI, daily activities, and caregiving received, was also collected in this survey. A total of 3091 questionnaires were sent, with an overall response rate of 47.6%. In this analysis, we excluded respondents with stage IV breast cancer and those missing responses for the FCRI‐SF inventory, resulting in a final sample of 1390 individuals.

Ethics approval for this study was received from the University of Chicago Institutional Review Board (Protocol number: 16352A). All participants provided written informed consent. This study followed the Strengthening the Reporting of Observational Studies in Epidemiology reporting guidelines for observational studies [13].

### 2.2 Outcome Measure: Fear of Cancer Recurrence

Our outcome of interest was FCR, which was assessed using the 9‐item FCRI‐SF, a widely validated screening tool [14, 15]. The FCRI‐SF defines recurrence as the possibility that cancer could return to the same site, or another part of the body; it evaluates the presence and severity of intrusive thoughts associated with FCR. For example, the first item asks respondents to rate how often they feel “worried or anxious about the possibility of cancer recurrence.” Each item is rated on a scale from 0 (not at all/never) to 4 (a great deal/all the time), producing a total score ranging from 0 to 36. Higher scores indicate more severe FCR. In this study, we examined the predictors of FCR both as a continuous score and a binary category. Recent data have postulated a cutoff of 22 for identifying clinically significant FCR according to clinical interview, with a reported sensitivity of 90% and specificity of 83.3% [15]. Adopting this cutoff, participants in our study were categorized into two groups: non‐csFCR (FCRI‐SF score < 22) and csFCR (FCRI‐SF score ≥ 22).

### 2.3 Sociodemographic, Clinical, and Treatment Factors

Sociodemographic characteristics were obtained from medical records and/or self‐reported cohort surveys. These included sex, race, and ethnicity, age at survey, marital status, annual income, educational attainment, body mass index (BMI), nativity status, primary language, English proficiency, religion, insurance type, alcohol consumption, and cigarette smoking. Race and ethnicity were self‐reported and categorized as Non‐Hispanic White, Non‐Hispanic Black, or Other. Other races/ethnicities included Hispanic, Asian American and Native Hawaiians/Pacific Islanders, Native American, and Alaska Natives. Cancer‐related clinical variables included age at diagnosis, family history of breast cancer, prior breast cancer recurrence, American Joint Committee on Cancer (AJCC) tumor stage, tumor grade, estrogen receptor (ER) status, progesterone receptor (PR) status, human epidermal growth factor receptor 2 (HER2) status, subtype, and history of another cancer diagnosis. Treatment‐related variables included receipt of chemotherapy, radiotherapy, hormonal therapy, or surgery. Chronic health conditions were collected from medical records and self‐reported via surveys, then categorized using the Charlson Comorbidity Index.

### 2.4 Stress and Care‐Related Factors

A stress score was calculated based on responses to 7 individual items adapted from validated item banks. These questions were adapted from the Patient‐Reported Outcomes Measurement Information System and a survey of COVID‐19–related risk perception and coping in the general population [16, 17]. The total score ranges from 0 to 28, and higher scores indicate higher stress. Further details on the development of this score are available in our previously published work [18, 19]. The stress score was assessed in 2020, 2021, and 2022 surveys and showed good internal consistency in all three waves of the survey (Cronbach's α = 0.83, 0.80, and 0.80, respectively). For this analysis, an individual's stress score was calculated as an average across all three timepoints. Furthermore, additional survey items assessed household composition (i.e., number of people living in the home) and social support, including whether the respondent needed care at home, and whether there were times when such care was needed but not received. A complete accounting of the temporal assessment of all variables used in this analysis can be found in Supporting Information S1: Table S1.

### 2.5 Physical Functioning

Physical functioning was measured using the Short Form‐36 (SF‐36) Physical Functioning subscale [20]. The SF‐36 questionnaire is a well‐established and widely used instrument for assessing health‐related quality of life. It has been extensively applied in both observational and randomized studies across a variety of health conditions and has been validated in diverse populations spanning different ages and demographic characteristics [21]. The questionnaire includes eight domains, including physical functioning. This domain evaluates limitations in ten mobility‐related activities, such as walking specific distances, carrying groceries, and performing basic self‐care tasks like bathing and dressing [20]. Scores on this subscale range from 0 to 100, with higher scores indicating better physical functioning and fewer physical limitations.

### 2.6 Statistical Analysis

Standard descriptive statistics were used to compare patient characteristics by csFCR status. For categorical variables, we tabulated counts and percentages and performed Chi‐squared tests or Fisher's Exact tests, where appropriate. To describe continuous data, we calculated means (SD) and compared the csFCR and non‐csFCR groups using *t*‐tests or Wilcoxon rank‐sum tests. Thereafter, multivariable logistic and linear regressions were fitted to determine the predictors of csFCR and higher FCR score, respectively. Variables that proved significant (*p* < 0.05) after adjusting for age were included in the multivariable analysis. We fitted several multivariable models. Our analytic approach was informed by Lazarus and Folkman's transactional model of stress and coping [11]. Using this framework, variables were entered sequentially in stepwise regression models as conceptually distinct domains influencing fear of cancer recurrence. Model 1 included baseline cancer‐related factors (age, ER status, and AJCC tumor stage). Model 2 added additional cancer events (prior recurrence and other malignancies). Model 3 incorporated receipt of care at home as an indicator of treatment context and available resources, and Model 4 further included physical functioning and stress as psychosocial and functional factors. This stepwise approach allowed us to assess the incremental contribution of each domain to the outcome. Adjusted odds ratios (aORs) and regression coefficients with corresponding 95% confidence intervals (CIs) were computed.

Approximately 22% of participants were missing data on the stress score, 11% on ER status, 5% on prior recurrence, and 4% on tumor stage. Data were occasionally missing for other variables included in the regression models. To avoid bias from listwise deletion, missing covariate data were addressed using multiple imputation by chained equations, assuming a missing at random mechanism [22]. The imputation model included all variables in Table 1 and FCRI‐SF score, except sex, English proficiency, insurance type, tumor subtype, tumor grade, and receipt of surgery. One hundred imputations were conducted, with 20 iterations of chained modeling to predict missing values. We combined results using Rubin's rule [23]. Imputed results were shown as the primary analyses. The complete case analyses using the non‐imputed data for csFCR and FCR scores are displayed in Supporting Information S1 Tables S2 and S3, respectively. All statistical tests were 2‐sided, with a *p*‐value of < 0.05 considered statistically significant. Analyses were performed using Stata, version 18 (StataCorp LLC, College Station, TX). Data were analyzed from January to June 2025.

**TABLE 1:** Demographic, socioeconomic, clinical, and psychosocial factors of patients with breast cancer, overall and by clinically significant FCR status.

Characteristic	Total	Non‐clinically significant FCR	Clinically significant FCR	*p*‐value^a^
	*N* = 1390	*n* = 1128	*n* = 262	
	No. (%)	No. (%)	No. (%)	
Age, mean (SD)	63.1 (11.7)	64.3 (11.3)	57.7 (12.0)	< 0.001
Years from diagnosis to survey, mean (SD)	9.1 (6.0)	9.3 (6.0)	8.3 (5.9)	0.014
BMI, mean (SD)	27.9 (6.0)	27.9 (6.2)	27.9 (6.0)	0.862
Sex				
Male	6 (0.4%)	5 (0.4%)	1 (0.4%)	1.000
Female	1384 (99.6%)	1123 (99.6%)	261 (99.6%)	
Race/ethnicity				
Non‐hispanic white	968 (69.6%)	779 (69.1%)	189 (72.1%)	0.388
Non‐hispanic black	309 (22.2%)	259 (23.0%)	50 (19.1%)	
Other	113 (8.1%)	90 (8.0%)	23 (8.8%)	
Education				
High school or lower	177 (14.5%)	151 (15.3%)	26 (11.2%)	0.287
Some college	223 (18.3%)	184 (18.6%)	39 (16.7%)	
Bachelor's degree	346 (28.4%)	273 (27.7%)	73 (31.3%)	
Graduate degree or above	474 (38.9%)	379 (38.4%)	95 (40.8%)	
Marital status				
Married or living with a partner	926 (67.3%)	739 (66.3%)	187 (71.6%)	0.162
Widowed	108 (7.9%)	93 (8.3%)	15 (5.7%)	
Divorced	141 (10.3%)	123 (11.0%)	18 (6.9%)	
Separated	24 (1.7%)	19 (1.7%)	5 (1.9%)	
Single or never married	176 (12.8%)	140 (12.6%)	36 (13.8%)	
Income				
Less than $49,999	105 (17.1%)	79 (16.2%)	26 (20.5%)	0.726
$50,000–$99,999	194 (31.5%)	160 (32.8%)	34 (26.8%)	
$100,000–$199,999	205 (33.3%)	159 (32.6%)	46 (36.2%)	
$200,000+	111 (18.0%)	90 (18.4%)	21 (16.5%)	
Nativity status				
Not US born	89 (9.0%)	75 (9.5%)	14 (7.1%)	0.283
US Born	897 (91.0%)	713 (90.5%)	184 (92.9%)	
Primary language				
English	93 (9.5%)	75 (9.6%)	18 (9.1%)	1.000
Other	888 (90.5%)	708 (90.4%)	180 (90.9%)	
English proficiency				
Average	9 (0.9%)	7 (0.9%)	2 (1.0%)	1.000
Medium	3 (0.3%)	3 (0.4%)	0 (0.0%)	
Well	965 (98.8%)	770 (98.7%)	195 (99.0%)	
Has religion				
No	180 (18.9%)	140 (18.4%)	40 (21.1%)	0.403
Yes	771 (81.1%)	621 (81.6%)	150 (78.9%)	
Insurance				
Not insured	2 (0.2%)	2 (0.2%)	0 (0.0%)	< 0.001
Private insurance	945 (72.7%)	738 (70.3%)	207 (83.1%)	
Medicaid	62 (4.8%)	49 (4.7%)	13 (5.2%)	
Medicare	269 (20.7%)	243 (23.1%)	26 (10.4%)	
Other insurance	21 (1.6%)	18 (1.7%)	3 (1.2%)	
Alcohol consumption				
Non‐drinker	601 (44.1%)	499 (45.2%)	102 (39.1%)	0.014
Previous drinker	188 (13.8%)	138 (12.5%)	50 (19.2%)	
Current drinker	575 (42.2%)	466 (42.2%)	109 (41.8%)	
Smoking				
Non‐smoker	891 (65.1%)	723 (65.3%)	168 (64.4%)	0.505
Previous smoker	399 (29.1%)	325 (29.3%)	74 (28.4%)	
Current smoker	79 (5.8%)	60 (5.4%)	19 (7.3%)	
Family history of breast cancer, 1st degree				
No	715 (71.9%)	577 (72.5%)	138 (69.3%)	0.378
Yes	280 (28.1%)	219 (27.5%)	61 (30.7%)	
Family history of breast cancer, 2nd degree				
No	694 (69.7%)	567 (71.2%)	127 (63.8%)	0.042
Yes	301 (30.3%)	229 (28.8%)	72 (36.2%)	
Tumor stage				
0	238 (17.9%)	203 (18.8%)	35 (13.9%)	0.040
I	622 (46.8%)	512 (47.5%)	110 (43.8%)	
II	342 (25.7%)	268 (24.9%)	74 (29.5%)	
III	127 (9.6%)	95 (8.8%)	32 (12.7%)	
Tumor grade				
1	177 (14.1%)	150 (14.8%)	27 (11.1%)	0.081
2	559 (44.5%)	459 (45.4%)	100 (41.0%)	
3	518 (41.3%)	401 (39.7%)	117 (48.0%)	
4	1 (0.1%)	1 (0.1%)	0 (0.0%)	
Estrogen receptor				
Negative	269 (21.6%)	225 (22.5%)	44 (18.0%)	0.129
Positive	975 (78.4%)	775 (77.5%)	200 (82.0%)	
Progesterone receptor				
Negative	413 (33.3%)	336 (33.7%)	77 (31.7%)	0.557
Positive	828 (66.7%)	662 (66.3%)	166 (68.3%)	
HER2 status				
Negative	857 (83.1%)	682 (83.1%)	175 (83.3%)	0.927
Positive	174 (16.9%)	139 (16.9%)	35 (16.7%)	
Tumor subtype				
HR+/HER2−	690 (67.0%)	544 (66.3%)	146 (69.5%)	0.265
HR+/HER2+	113 (11.0%)	86 (10.5%)	27 (12.9%)	
HR−/HER2+	61 (5.9%)	53 (6.5%)	8 (3.8%)	
TNBC	166 (16.1%)	137 (16.7%)	29 (13.8%)	
Had surgery				
None	8 (0.6%)	6 (0.6%)	2 (0.8%)	0.807
Lumpectomy	694 (52.2%)	567 (52.8%)	127 (49.8%)	
Mastectomy	470 (35.4%)	377 (35.1%)	93 (36.5%)	
Bilateral mastectomy	157 (11.8%)	124 (11.5%)	33 (12.9%)	
Chemotherapy				
No	738 (55.2%)	616 (57.0%)	122 (47.7%)	0.007
Yes	598 (44.8%)	464 (43.0%)	134 (52.3%)	
Radiotherapy				
No	508 (38.1%)	415 (38.6%)	93 (36.3%)	0.507
Yes	824 (61.9%)	661 (61.4%)	163 (63.7%)	
Hormone therapy				
No	447 (33.5%)	369 (34.2%)	78 (30.5%)	0.260
Yes	889 (66.5%)	711 (65.8%)	178 (69.5%)	
Other cancer				
None	1185 (86.0%)	973 (87.0%)	212 (81.9%)	0.016
Skin cancer other than melanoma	55 (4.0%)	46 (4.1%)	9 (3.5%)	
Other cancer excluding non‐melanoma skin cancers	138 (10.0%)	100 (8.9%)	38 (14.7%)	
Prior recurrence status				
Disease‐free	1274 (96.2%)	1041 (97.1%)	233 (92.5%)	< 0.001
Recurrence	50 (3.8%)	31 (2.9%)	19 (7.5%)	
Comorbidity index				
0	1136 (86.2%)	915 (85.9%)	221 (87.4%)	0.576
1	87 (6.6%)	74 (6.9%)	13 (5.1%)	
2+	95 (7.2%)	76 (7.1%)	19 (7.5%)	
SF‐36 physical functioning, mean (SD)	90.3 (10.6)	90.7 (10.4)	88.5 (11.3)	0.003
Number of people living with, mean (SD)	1.7 (1.5)	1.6 (1.5)	1.8 (1.3)	0.202
Received care at home				
Did not need care	1009 (73.4%)	842 (75.4%)	167 (64.7%)	< 0.001
Needed care and received it	217 (15.8%)	177 (15.8%)	40 (15.5%)	
Needed care and did not receive it	37 (2.7%)	24 (2.1%)	13 (5.0%)	
Other^b^	112 (8.1%)	74 (6.6%)	38 (14.7%)	
Stress score, mean (SD)	9.0 (4.1)	8.3 (3.7)	12.0 (4.6)	< 0.001

Abbreviations: BMI, body mass index; FCR, fear of cancer recurrence; HER2, human epidermal growth factor receptor‐2; HR, hormone receptor; No., number; SD, standard deviation; TNBC, triple‐negative breast cancer.

^a^ *p* value was computed using *t*‐tests, Fisher's exact tests, Person's Chi‐squared tests, or Wilcoxon Rank‐Sum Tests, as appropriate.

^b^ The “other” category includes individuals whose responses do not fit neatly into the first three predefined groups (e.g., individuals who both received care and had unmet needs or individuals with incomplete, contradictory or conflicting patterns).

## 3 Results

### 3.1 Participant Characteristics

The study included 1390 participants, with a mean (SD) age of 63.1 (11.7) years and a mean (SD) time since diagnosis to survey of 9.1 (6.0) years. Overall, 262 (18.8%) survey respondents were classified as having csFCR (Table 1). The mean (SD) FCR scores in this sample were 14.00 (7.57).

### 3.2 Demographic and Lifestyle Characteristics

**FIGURE 1:**
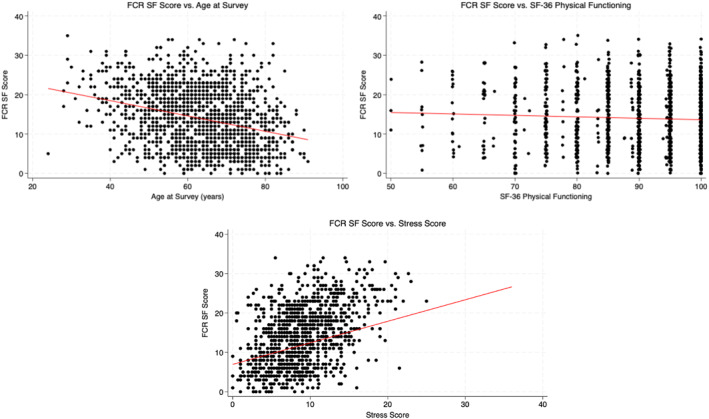
Distributions of fear of cancer recurrence (FCR) scores by age at survey, SF‐36 physical functioning, and stress score.

Participants with csFCR were younger (mean age: 57.7 vs. 64.3 years, *p* < 0.001) and had a shorter follow‐up time since diagnosis (8.3 vs. 9.3 years, *p* = 0.014) compared to the non‐csFCR group (Table 1). As illustrated in Figure 1, there was a strong negative association between FCR score and age at survey (*p* < 0.001). Significant differences were observed in insurance status (*p* < 0.001), with the csFCR group having higher percentages of private insurance (83.1% vs. 70.3%) and lower percentages of Medicare (10.4% vs. 23.1%). Alcohol use also differed (*p* = 0.014), with the csFCR group reporting more previous drinkers (19.2% vs. 12.5%) and fewer non‐drinkers (39.1% vs. 45.2%). No significant differences were found in BMI, sex, race/ethnicity, educational attainment, marital status, income, nativity status, language proficiency, religion, and smoking (all *p* values > 0.05) (Table 1).

### 3.3 Clinical and Treatment‐Related Factors

The csFCR group had higher percentage of chemotherapy use (52.3% vs. 43.0%, *p* = 0.007), prior recurrence (7.5% vs. 2.9%, *p* < 0.001), and other cancers excluding non‐melanoma skin cancer (14.7% vs. 8.9%, *p* = 0.016) (Table 1). Among the 50 participants who had a prior recurrence, 17 (34.0%) had a distant recurrence, 17 (34.0%) had a local recurrence, 9 (18.0%) had a regional recurrence, and 7 (14.0%) had an in situ recurrence. Mean FCR scores differed significantly between the groups (*p* = 0.002) and were highest in the distant recurrence group (19.6), followed by local recurrence (18.2), in situ recurrence (16.6), regional recurrence (15.6), and then disease‐free (13.8) (Supporting Information S1: Table S4). AJCC tumor stage at diagnosis was also significantly different between the csFCR and non‐csFCR groups (*p* = 0.040), with a higher proportion of stage 2 and 3 tumors in the csFCR group. No significant differences were observed in tumor grade, ER status, PR status, HER2 status, tumor subtype, receipt of surgery, radiotherapy, hormone therapy, or comorbidity index (Table 1).

**FIGURE 2:**
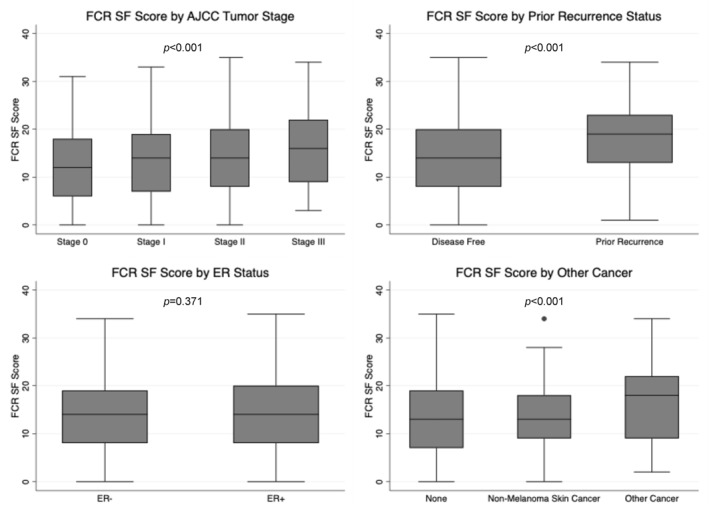
Distributions of fear of cancer recurrence (FCR) scores by tumor stage, prior recurrence, ER status, and other cancer.

Participants with stage III disease had the highest mean FCRI‐SF score (15.7 [SD, 7.9]), followed by those with stage II (14.5 [SD, 7.8]), stage I (13.8 [SD, 7.5]), and stage 0 (12.4 [SD, 7.1]; *p* < 0.001). Similarly, participants with a previous recurrence reported significantly higher scores than those without (18.0 [SD, 8.1] vs. 13.8 [SD, 7.5]; *p* < 0.001). Those with another malignancy (excluding non‐melanoma skin cancer) also had higher FCR scores compared to those with non‐melanoma skin cancer and those with no other cancers (16.3 [SD, 7.7], 14.1 [SD, 7.2] vs. 13.7 [SD, 7.5]; *p* < 0.001). No significant differences in FCRI‐SF scores by ER status were observed (Figure 2).

### 3.4 Physical Functioning, Stress, and Care‐Related Factors

Participants with csFCR reported lower physical functioning (SF‐36 mean: 88.5 [SD, 11.3] vs. 90.7 [SD, 10.4]; *p* = 0.003) and increased stress (12.0 [SD, 4.6] vs. 8.3 [SD, 3.7]; *p* < 0.001). In addition, a greater proportion of people in the csFCR group reported unmet care needs at home, including needing care and not receiving it (5.0% vs. 2.1%) and other forms of unmet care needs (14.7% vs. 6.6%) (*p* < 0.001) (Table 1). FCRI‐SF scores were moderately associated with physical functioning (*p* = 0.055) and were strongly associated with stress score (*p* < 0.001) (Figure 1).

### 3.5 Multivariable Analysis of Factors for Clinically Significant FCR

Table 2 presents the logistic regression results for predictors of csFCR using the imputed dataset. In the fully adjusted model (Model 4), participants with a history of other malignancies (excluding non‐melanoma skin cancer) had significantly higher odds of csFCR (aOR, 2.64; 95% CI: 1.66–4.21; *p* < 0.001). Those with only non‐melanoma skin cancer had no increased odds (aOR, 1.26; 95% CI: 0.60–2.84; *p* = 0.576). A history of cancer recurrence was also associated with elevated odds of csFCR (aOR, 2.26; 95% CI: 1.14–4.47; *p* = 0.019). Stress was a strong predictor; each one SD increase was associated with over twice the odds of csFCR (aOR, 2.40; 95% CI: 1.98–2.91; *p* < 0.001). Participants with ER‐positive tumors had higher odds compared to those with ER‐negative tumors (aOR, 1.60; 95% CI: 1.06–2.40; *p* = 0.025). Older age was inversely associated with csFCR (aOR, 0.64 per 10‐year increase; 95% CI: 0.56–0.73; *p* < 0.001).

**TABLE 2:** Association between various factors and clinically significant fear of cancer recurrence in patients with breast cancer.

Characteristic	Model 1		Model 2		Model 3		Model 4	
	aOR (95% CI)	*p*‐value	aOR (95% CI)	*p*‐value	aOR (95% CI)	*p*‐value	aOR (95% CI)	*p*‐value
Age at survey^a^	0.62 (0.55, 0.70)	< 0.001	0.60 (0.53, 0.68)	< 0.001	0.59 (0.52, 0.66)	< 0.001	0.64 (0.56, 0.73)	< 0.001
Estrogen receptor								
Negative	Reference		Reference		Reference		Reference	
Positive	1.67 (1.14, 2.42)	0.008	1.67 (1.14, 2.44)	0.008	1.66 (1.13, 2.44)	0.009	1.60 (1.06, 2.40)	0.025
Tumor stage								
0	Reference		Reference		Reference		Reference	
I	1.23 (0.81, 1.87)	0.328	1.21 (0.80, 1.84)	0.373	1.24 (0.81, 1.90)	0.323	1.37 (0.87, 2.16)	0.170
II	1.50 (0.96, 2.36)	0.076	1.52 (0.96, 2.40)	0.072	1.56 (0.98, 2.48)	0.059	1.43 (0.87, 2.35)	0.156
III	1.89 (1.08, 3.28)	0.025	1.83 (1.05, 3.21)	0.034	1.77 (1.00, 3.14)	0.050	1.81 (0.97, 3.38)	0.061
	*Linear trend test p =* 0.012		*Linear trend test p = 0.015*		*Linear trend test p = 0.020*		*Linear trend test p = 0.072*	
Other cancer								
None	Reference		Reference		Reference		Reference	
Skin cancer other than melanoma	NA	NA	1.31 (0.61, 2.78)	0.486	1.31 (0.61, 2.82)	0.486	1.26 (0.60, 2.84)	0.576
Other cancer excluding non‐melanoma skin cancers	NA	NA	2.33 (1.52, 3.57)	< 0.001	2.28 (1.48, 3.52)	< 0.001	2.64 (1.66, 4.21)	< 0.001
Prior recurrence status								
Disease‐free	Reference		Reference		Reference		Reference	
Recurrence	NA	NA	2.38 (1.28, 4.41)	0.006	2.30 (1.23, 4.30)	0.009	2.26 (1.14, 4.47)	0.019
Received care at home								
Did not need care	Reference		Reference		Reference		Reference	
Needed care and got it	NA	NA	NA	NA	1.21 (0.81, 1.81)	0.356	0.72 (0.45, 1.13)	0.151
Needed care and did not get it	NA	NA	NA	NA	2.79 (1.34, 5.82)	0.006	1.27 (0.54, 2.99)	0.585
Other	NA	NA	NA	NA	2.96 (1.88, 4.68)	< 0.001	1.38 (0.82, 2.32)	0.230
SF‐36 physical functioning^b^	NA	NA	NA	NA	NA	NA	0.89 (0.75, 1.05)	0.166
Stress score^b^	NA	NA	NA	NA	NA	NA	2.40 (1.98, 2.91)	< 0.001

Abbreviations: aOR, adjusted odds ratio; CI, confidence interval; NA, not applicable.

^a^ The aORs and 95% CIs were per 10‐year increase.

^b^ The aORs and 95% CIs were per 1 standard deviation increase.

Across models 1–3, we observed a significant monotonic trend in the odds of csFCR with advanced stage (*p* < 0.05), but this became marginally significant in Model 4 after adjusting for physical function and stress (*p* = 0.072). Needing care at home and not receiving it (aOR, 2.79; 95% CI: 1.34–5.82; *p* = 0.006) or having other unmet care needs at home (aOR, 2.96; 95% CI: 1.88–4.68; *p* < 0.001) was also associated with csFCR in Model 3; however, this was not significant after adjustment in the final model. Physical functioning was not associated with csFCR after adjusting for all other variables included in Model 4. These results are consistent with the complete case analysis, with the exception of ER‐positive disease, which was non‐significant after adjustment for physical functioning and stress (Supporting Information S1: Table S2).

### 3.6 Multivariable Analysis of Factors Correlated With FCR Severity

Table 3 describes the linear regression models examining factors associated with FCRI‐SF score using the imputed dataset. In the final model, FCR scores were strongly associated with having another cancer (excluding non‐melanoma skin cancer), with those participants reporting FCR scores 3.55 higher than those with no other cancers (95% CI: 2.38–4.59; *p* < 0.001). Similarly, those with a prior recurrence reported FCR scores 2.73 points higher than those who remained disease‐free (95% CI: 0.86–4.59; *p* = 0.004). Additionally, stress was associated with higher FCR scores, with each 1‐SD increase in stress score corresponding to a 3.17‐point rise in FCR severity (95% CI: 2.76–3.58; *p* < 0.001). Advanced tumor stage also contributed significantly, with stage I (+1.65 points), stage II (+1.56 points), and stage III (+2.72 points) tumors demonstrating progressively higher FCR scores compared to stage 0 (*p*‐trend = 0.001). Participants with ER‐positive breast cancer reported FCR scores 1.10 higher than ER‐negative disease (95% CI: 0.020–1.99; *p* = 0.017). Older age remained a strong protective factor, as each 10‐year increase in age was linked to a 1.55‐point reduction in FCR scores (95% CI: −1.87 to −1.23; *p* < 0.001). Having other care‐related challenges at home, which showed significant associations in earlier models (model 3), lost significance in the final analysis (*p* = 0.127) (model 4). Physical functioning and having skin cancer (excluding melanoma) were not associated with FCR severity in the final model. These results are consistent with the complete case analysis, with the exception of ER‐positive disease, which became non‐significant after adjustment for physical functioning and stress (Supporting Information S1: Table S3).

**TABLE 3:** Association between various factors and fear of cancer recurrence score in patients with breast cancer.

Characteristic	Model 1		Model 2		Model 3		Model 4	
	Coef. (95% CI)	*p*‐value	Coef. (95% CI)	*p*‐value	Coef. (95% CI)	*p*‐value	Coef. (95% CI)	*p*‐value
Age at survey^a^	−1.93 (−2.26, −1.61)	< 0.001	−2.05 (−2.37, −1.72)	< 0.001	−2.11 (−2.43, −1.79)	< 0.001	−1.55 (−1.87, −1.23)	< 0.001
Estrogen receptor								
Negative	Reference		Reference		Reference		Reference	
Positive	1.43 (0.45, 2.41)	0.004	1.43 (0.47, 2.40)	0.004	1.42 (0.46, 2.38)	0.004	1.10 (0.20, 1.99)	0.017
Stage								
0	Reference		Reference		Reference		Reference	
I	1.36 (0.29, 2.43)	0.013	1.28 (0.22, 2.33)	0.018	1.29 (0.25, 2.34)	0.015	1.65 (0.68, 2.62)	0.001
II	1.69 (0.49, 2.90)	0.006	1.74 (0.55, 2.93)	0.004	1.75 (0.57, 2.92)	0.004	1.56 (0.46, 2.66)	0.005
III	2.98 (1.41, 4.55)	< 0.001	2.87 (1.32, 4.41)	< 0.001	2.67 (1.13, 4.20)	0.001	2.72 (1.29, 4.14)	< 0.001
	*Linear trend test p < 0.001*		*Linear trend test p < 0.001*		*Linear trend test p < 0.001*		*Linear trend test p =* 0.001	
Other cancer								
None	Reference		Reference		Reference		Reference	
Skin cancer other than melanoma	NA	NA	1.69 (−0.26, 3.63)	0.089	1.69 (−0.23, 3.62)	0.084	1.52 (−0.24, 3.29)	0.091
Other cancer excluding non‐melanoma skin cancers	NA	NA	3.57 (2.31, 4.84)	< 0.001	3.38 (2.12, 4.64)	< 0.001	3.55 (2.38, 4.72)	< 0.001
Prior recurrence status								
Disease‐free	Reference		Reference		Reference		Reference	
Recurrence	NA	NA	3.31 (1.31, 5.32)	0.001	3.18 (1.20, 5.16)	0.002	2.73 (0.86, 4.59)	0.004
Received care at home								
Did not need care	Reference		Reference		Reference		Reference	
Needed care and got it	NA	NA	NA	NA	1.12 (0.07, 2.17)	0.037	−0.41 (−1.44, 0.62)	0.435
Needed care and did not get it	NA	NA	NA	NA	1.95 (−0.38, 4.29)	0.101	−0.70 (−2.92, 1.52)	0.536
Other	NA	NA	NA	NA	3.77 (2.38, 5.16)	< 0.001	1.06 (−0.30, 2.43)	0.127
SF‐36 physical functioning^b^	NA	NA	NA	NA	NA	NA	−0.09 (−0.49, 0.31)	0.667
Stress score^b^	NA	NA	NA	NA	NA	NA	3.17 (2.76, 3.58)	< 0.001

Abbreviations: CI, confidence interval; Coef., coefficient; NA, not applicable.

^a^ The coefficients and 95% CIs were per 10‐year increase.

^b^ The coefficients and 95% CIs were per 1 standard deviation increase.

## 4 Discussion

In this study of a large multiethnic cohort of breast cancer survivors, we found that approximately 1 in 5 respondents experienced clinically significant levels of FCR using a validated and comprehensive measure. We also identified key clinical, psychosocial, and demographic factors associated with csFCR and FCR severity.

Consistent with prior studies, younger patients were at higher risk for both csFCR and increased FCR scores [24]. This may be attributable to the disruption of life‐stage responsibilities after a cancer diagnosis, such as caregiving, parenting, and concurrent financial obligations [24]. Additionally, this study demonstrated that prior recurrence was associated with over a two‐fold increase in the odds of csFCR and significantly higher FCR scores. This likely results from heightened vigilance and uncertainty experienced by patients who have already faced disease progression [25]. Individuals who have had a prior recurrence are at a high risk for future recurrence and may remain hyper‐aware of bodily symptoms or potential warning signs, intensifying their fear responses [26]. Prior experiences of recurrence may also reduce confidence in treatment efficacy or long‐term remission, compounding emotional distress and fear of another recurrence [27]. This finding is consistent with most studies in which recurrence or metastatic diagnosis was significantly associated with FCR [28].

Moreover, we found that breast cancer patients with a history of other malignancies (excluding non‐melanoma skin cancer) had significantly higher odds of experiencing csFCR and greater FCR severity. This may result from heightened health vigilance, cumulative trauma from multiple cancer diagnoses, or increased uncertainty about future health [29]. Patients with more than one cancer diagnosis may also face more intensive medical surveillance, which can serve as a reminder of disease vulnerability and increase persistent fears [29]. In contrast, patients with a history of non‐melanoma skin cancer had similar FCR to those with no prior cancer diagnoses. This aligns with prior research indicating that non‐melanoma skin cancers, which typically require minimal treatment and pose limited threat to long‐term health, are associated with low levels of cancer‐related worry and risk of recurrence [30]. In addition, we found that ER‐positive disease was significantly associated with csFCR and higher FCR scores in the multivariable model. This association may be a result of higher rates of long‐term recurrence compared to ER‐negative tumors, particularly beyond 5 years, among patients with ER‐positive breast cancer [31, 32]. Given that the average follow‐up years since diagnosis in our sample were 9.1 years, it is plausible that this subgroup of patients was entering the period of continued risk for late recurrence, therefore contributing to increased FCR. Taken together, these findings warrant tailored psychosocial support in survivorship care.

In this analysis, tumor stage at diagnosis demonstrated a consistent linear trend with FCR severity and a marginally significant trend with csFCR, indicating that more advanced disease may heighten long‐term fears of recurrence. Studies investigating whether FCR varies by tumor stage have been inconclusive, with some reporting no difference in FCR by tumor stage and others finding strong associations [33, 34]. In breast cancer, tumor stage is a prognostic indicator used to assess the likelihood of survival and recurrence risk [35]. Higher‐stage cancers often require more aggressive treatment and may be associated with increased risk of recurrence. As a result, patients with advanced‐stage tumors may perceive a greater threat of recurrence, experience heightened psychological distress, and feel less confident in the long‐term treatment efficacy. This psychological burden may, in turn, elevate FCR. The consistent trend and the interplay between stage and perceived risk of recurrence are worth exploring in future research.

Stress also had a strong association with both csFCR and FCR score, even after controlling for other covariates. Each SD increase in stress was associated with approximately a two‐and‐a‐half‐fold increase in the odds of csFCR and a three‐point increase in FCR score. Stress may exacerbate intrusive thoughts, uncertainty about the future, and emotional vulnerability, all of which are central components of FCR [36]. Persistent stress can also impair emotional regulation and hinder the development of adaptive coping strategies [37]. Furthermore, stress may serve as a barrier to accessing healthcare resources, psychosocial support services, or educational materials that could otherwise help manage concerns about recurrence [38]. These findings support existing evidence that managing stress is essential for fostering emotional resilience during survivorship [39]. Interventions that reduce stress, such as peer support groups, survivorship mentorship programs, or structured group therapy, may be valuable in reducing FCR. Integrating social support assessments into routine survivorship care could help identify vulnerable individuals and tailor interventions accordingly. Moreover, these results align with Lazarus and Folkman's transactional model of stress and coping. In our analyses, baseline disease characteristics and other cancer events may represent objective stressors, while stress reflects an individual's host factor shaping appraisal and coping responses. The strong association between stress and FCR in our models may be indicative of the role of appraisal processes in influencing survivorship experiences.

Interestingly, while having unmet care needs at home and physical functioning were initially associated with csFCR, these associations were attenuated in multivariable models. These patterns suggest that while having unmet care needs and physical functioning provide important context, they may be insufficient on their own to explain which patients experience the most distressing or functionally impairing levels of csFCR. Additionally, in contrast to some prior studies examining factors associated with FCR in other cancer types, we did not find significant associations between FCR and most demographic, socioeconomic, or lifestyle characteristics, including BMI, marital status, race/ethnicity, education, religion, insurance, or smoking [7]. These findings suggest that FCR is universal among breast cancer survivors regardless of demographic characteristics, and psychosocial interventions for FCR should be targeted at all breast cancer patients.

### 4.1 Implications

The findings of this study have several important clinical implications. Routine screening for FCR, particularly among younger patients, those reporting stress, recurrent disease, other malignancies, or ER‐positive disease, may help identify individuals at greatest risk. Interventions aimed at improving social support, psychological well‐being, and functional status may be effective strategies to reduce the burden of FCR in survivorship care. Psychological interventions, such as cognitive behavioral therapy, acceptance and commitment therapy, and mind‐body interventions, are effective in reducing FCR in patients with adult‐onset cancer [40, 41]. Moreover, integrating psychosocial assessment into follow‐up oncology care can facilitate timely referral to counseling or support services. Finally, these findings suggest that health care professionals should routinely assess FCR as a part of providing comprehensive care to breast cancer patients and survivors. The FCRI‐SF is well‐suited for rapid administration and interpretation within clinical settings [14, 15].

Additionally, our findings highlight the need for professional organizations, such as the American Society of Clinical Oncology, to establish clinical guidelines for the assessment and management of FCR in survivorship care. Particular attention is needed for younger breast cancer survivors, who are disproportionately affected by FCR and represent a growing population due to rising incidence rates in younger women. As treatment strategies evolve, future studies should also examine how treatment de‐escalation (e.g., shorter durations of therapy) and active surveillance influence FCR perceptions. A better understanding of how these emerging approaches impact emotional well‐being will be essential to guiding shared decision‐making and optimizing long‐term quality of life in breast cancer survivorship.

### 4.2 Limitations

Several limitations of this study should be considered. First, the response rate (47.6%) may limit the representativeness of the sample. Given that FCR Scores were significantly associated with age, it is possible that selection bias occurred if non‐respondents differed systematically in age from respondents. For instance, the prevalence of csFCR may be underestimated if non‐respondents were younger. Although the response rate was 47.6%, the demographic and clinical characteristics of non‐respondents were generally similar to those of respondents, reducing the likelihood of significant non‐response bias (Supporting Information S1: Table S5). Second, the cross‐sectional design for variables assessed in 2023 (e.g., age at survey, caregiving received, and physical functioning) precludes conclusions about the temporal trajectory or causality of FCR among breast cancer patients. However, several key variables (e.g., tumor stage, ER status, prior recurrence, history of another cancer, and stress scores) were collected before 2023, allowing for partial temporal ordering. As such, the design reflects some cohort characteristics, especially for variables assessed before FCR. Third, due to the lack of available data, we were unable to assess pre‐diagnosis anxiety as a potential predictor, even though it may play a significant role in the development of FCR [10]. Despite these limitations, the study provides important insight into key demographic, psychosocial, and clinical correlates of FCR in a multiethnic cohort of patients with breast cancer.

## 5 Conclusion

In summary, FCR remains a significant concern among long‐term breast cancer survivors. Younger age, stress, disease recurrence, having other malignancies, and ER‐positive disease are the strongest predictors of csFCR and FCR severity. Our findings help lay the groundwork for an improved understanding of FCR among diverse breast cancer populations, which is vital to informing screening strategies and intervention efforts.

## Author Contributions

**Armaan Jamal:** methodology, data curation, formal analysis, visualization, writing – original draft, writing – review and editing. **Fangyuan Zhao:** conceptualization, investigation, data curation, writing – review and editing. **Jincong Q. Freeman:** conceptualization, investigation, data curation, writing – review and editing. **Yijia Sun:** data curation, writing – review and editing. **Marcia M. Tan:** investigation, supervision, writing – review and editing. **Rita Nanda:** conceptualization, investigation, supervision, writing – review and editing. **Nan Chen:** conceptualization, investigation, supervision, writing – review and editing. **Olufunmilayo I. Olopade:** conceptualization, investigation, supervision, funding acquisition, writing – review and editing. **Dezheng Huo:** conceptualization, methodology, investigation, data curation, supervision, funding acquisition, writing – review and editing.

## Conflicts of Interest

Dr. Nanda has disclosed advisory board involvement with and research funding from Arvinas, AstraZeneca, BeyondSpring, Celgene, FujiFilm, Genentech/Roche, Gilead, Infinity, iTeos, Merck, OBI Pharma, OncoSec, Pfizer, Relay Therapeutics, SeaGen, Sun Pharma, and Taiho. Dr. Chen has disclosed consulting with Seagen, Guardant Health, Novartis, AstraZeneca, Daiichi Sankyo, Stemline, and research funding from Olema, Puma, and Merck. Dr. Olopade has disclosed financial relationships with CancerIQ and TempusAI and research funding from Color Genomics and Roche/Genentech. All other authors report no conflicts of interest.

## Supporting information


Supporting Information S1
