# Supplementary material for: Factors Associated With Fear of Cancer Recurrence in a Multiethnic Cohort of Patients With Breast Cancer
Source: Psychooncology. 2025 Oct 16;34(10):e70307. doi: 10.1002/pon.70307 (PMC12531357; doi:10.1002/pon.70307)
Supplement: Supplementary file 1 — Supporting Information S1 [file PON-34-e70307-s001.docx]

**Supplemental Table S1. Temporal Assessment of Key Factors Used in this Study**

| **Characteristic** | **At Diagnosis** | Any point before 2023 | **2020** | **2021** | **2022** | **2023** |
| --- | --- | --- | --- | --- | --- | --- |
| Age at survey |  |  |  |  |  | X |
| Years from diagnosis to survey | X |  |  |  |  | X |
| BMI |  |  |  |  |  | X |
| Sex | X |  |  |  |  |  |
| Race/Ethnicity | X |  |  |  |  |  |
| Education | X |  |  |  |  |  |
| Marital Status | X |  |  |  |  |  |
| Income |  |  |  | X |  |  |
| Nativity Status | X |  |  |  |  |  |
| Primary Language | X |  |  |  |  |  |
| English Proficiency | X |  |  |  |  |  |
| Has Religion | X |  |  |  |  |  |
| Insurance | X |  |  |  |  |  |
| Alcohol Consumption | X |  |  |  |  |  |
| Smoking | X |  |  |  |  |  |
| Family History of Breast Cancer, 1st Degree | X |  |  |  |  |  |
| Family History of Breast Cancer, 2nd Degree | X |  |  |  |  |  |
| Tumor Stage | X |  |  |  |  |  |
| Tumor Grade | X |  |  |  |  |  |
| Estrogen Receptor | X |  |  |  |  |  |
| Progesterone Receptor | X |  |  |  |  |  |
| HER2 Status | X |  |  |  |  |  |
| Tumor Subtype | X |  |  |  |  |  |
| Had Surgery | X^†^ |  |  |  |  |  |
| Chemotherapy | X^†^ |  |  |  |  |  |
| Radiotherapy | X^†^ |  |  |  |  |  |
| Hormone Therapy | X^†^ |  |  |  |  |  |
| Other Cancer |  | X |  |  |  |  |
| Prior Recurrence Status |  | X |  |  |  |  |
| Comorbidity Index | X |  |  |  |  |  |
| SF-36 Physical Functioning |  |  |  |  |  | X |
| Number of People Living With |  |  |  |  |  | X |
| Received Care at Home |  |  |  |  |  | X |
| Stress Score |  |  | X | X | X |  |

^†^Treatment was assessed at diagnosis or shortly after.

**Supplemental Table S2. Complete Case Analysis of the Association between Various Factors and Clinically Significant Fear of Cancer Recurrence among Breast Cancer Patients**

| **Characteristic** | **Model 1 (N = 1,239)** | | **Model 2 (N = 1,218)** | | **Model 3 (N = 1,204)** | | **Model 4 (N = 949)** | |
| --- | --- | --- | --- | --- | --- | --- | --- | --- |
|  | **aOR (95% CI)** | ***p*-value** | **aOR (95% CI)** | ***p*-value** | **aOR (95% CI)** | ***p*-value** | **aOR (95% CI)** | ***p*-value** |
| **Age at Survey** ^†^ | 0.60 (0.53, 0.68) | <0.001 | 0.59 (0.51, 0.67) | <0.001 | 0.57 (0.50, 0.65) | <0.001 | 0.62 (0.53, 0.73) | <0.001 |
| **Estrogen Receptor** |  |  |  |  |  |  |  |  |
| Negative | Reference |  | Reference |  | Reference |  | Reference |  |
| Positive | 1.64 (1.12, 2.40) | 0.011 | 1.61 (1.09, 2.38) | 0.016 | 1.58 (1.06, 2.36) | 0.025 | 1.36 (0.86, 2.15) | 0.184 |
| **Stage** |  |  |  |  |  |  |  |  |
| 0 | Reference |  | Reference |  | Reference |  | Reference |  |
| I | 1.17 (0.73, 1.87) | 0.510 | 1.08 (0.67, 1.74) | 0.751 | 1.11 (0.68, 1.79) | 0.684 | 1.29 (0.74, 2.26) | 0.372 |
| II | 1.41 (0.86, 2.32) | 0.178 | 1.35 (0.81, 2.24) | 0.247 | 1.36 (0.81, 2.28) | 0.244 | 1.26 (0.68, 2.32) | 0.460 |
| III | 1.75 (0.97, 3.16) | 0.065 | 1.63 (0.89, 2.97) | 0.113 | 1.54 (0.83, 2.86) | 0.172 | 1.45 (0.69, 3.07) | 0.326 |
|  | *Linear trend test p =* 0.035 | | *Linear trend test p = 0.051* | | *Linear trend test p = 0.089* | | *Linear trend test p = 0.414* | |
| **Other Cancer** |  |  |  |  |  |  |  |  |
| None | Reference |  | Reference |  | Reference |  | Reference |  |
| Skin Cancer Other than Melanoma | NA | NA | 1.19 (0.51, 2.76) | 0.687 | 1.22 (0.52, 2.86) | 0.641 | 1.31 (0.50, 3.42) | 0.584 |
| Other Cancer Excluding Non-Melanoma Skin Cancers | NA | NA | 2.39 (1.51, 3.80) | <0.001 | 2.35 (1.45, 3.78) | <0.001 | 3.12 (1.78, 5.48) | <0.001 |
| **Prior Recurrence Status** |  |  |  |  |  |  |  |  |
| Disease Free | Reference |  | Reference |  | Reference |  | Reference |  |
| Recurrence | NA | NA | 2.49 (1.18, 5.24) | 0.016 | 2.43 (1.15, 5.17) | 0.021 | 4.59 (1.77, 11.93) | 0.002 |
| **Received Care at Home** |  |  |  |  |  |  |  |  |
| Did Not Need Care | Reference |  | Reference |  | Reference |  | Reference |  |
| Needed Care and Got It | NA | NA | NA | NA | 1.15 (0.74, 1.78) | 0.527 | 0.62 (0.36, 1.08) | 0.093 |
| Needed Care and Did Not Get It | NA | NA | NA | NA | 3.14 (1.45, 6.82) | 0.004 | 2.25 (0.85, 5.95) | 0.102 |
| Other | NA | NA | NA | NA | 3.05 (1.86, 4.99) | <0.001 | 1.49 (0.79, 2.83) | 0.220 |
| **SF-36 Physical Functioning** ^‡^ | NA | NA | NA | NA | NA | NA | 0.84 (0.69, 1.03) | 0.087 |
| **Stress Score** ^‡^ | NA | NA | NA | NA | NA | NA | 2.31 (1.88, 2.84) | <0.001 |

Abbreviations: aOR, adjusted odds ratio; CI, confidence interval; NA, not applicable.

^†^ The aORs and 95% CIs were per 10-year increase.

^‡^ The aORs and 95% CIs were per 1 standard deviation increase.

**Supplemental Table S3. Complete Case Analysis of the Association between Various Factors and Fear of Cancer Recurrence Score among Breast Cancer Patients**

| **Characteristic** | **Model 1 (N = 1,239)** | | **Model 2 (N = 1,218)** | | **Model 3 (N = 1,204)** | | **Model 4 (N = 949)** | |
| --- | --- | --- | --- | --- | --- | --- | --- | --- |
|  | **Coef. (95% CI)** | ***p*-value** | **Coef. (95% CI)** | ***p*-value** | **Coef. (95% CI)** | ***p*-value** | **Coef. (95% CI)** | ***p*-value** |
| **Age at Survey** ^†^ | -2.10 (-2.44, -1.76) | <0.001 | -2.19 (-2.54, -1.84) | <0.001 | -2.26 (-2.60, -1.92) | <0.001 | -1.70 (-2.08, -1.32) | <0.001 |
| **Estrogen Receptor** |  |  |  |  |  |  |  |  |
| Negative | Reference |  | Reference |  | Reference |  | Reference |  |
| Positive | 1.26 (0.26, 2.26) | 0.013 | 1.29 (0.29, 2.29) | 0.011 | 1.30 (0.30, 2.29) | 0.011 | 0.89 (-0.11, 1.90) | 0.082 |
| **Stage** |  |  |  |  |  |  |  |  |
| 0 | Reference |  | Reference |  | Reference |  | Reference |  |
| I | 1.55 (0.32, 2.79) | 0.014 | 1.29 (0.06, 2.52) | 0.039 | 1.31 (0.10, 2.52) | 0.034 | 2.09 (0.86, 3.33) | 0.001 |
| II | 1.78 (0.43, 3.13) | 0.010 | 1.72 (0.38, 3.06) | 0.012 | 1.70 (0.37, 3.03) | 0.012 | 1.94 (0.58, 3.30) | 0.005 |
| III | 3.00 (1.33, 4.67) | <0.001 | 2.78 (1.11, 4.45) | 0.001 | 2.59 (0.93, 4.24) | 0.002 | 2.89 (1.17, 4.61) | 0.001 |
|  | *Linear trend test p =* 0.001 | | *Linear trend test p = 0.001* | | *Linear trend test p =* 0.002 | | *Linear trend test p =* 0.005 | |
| **Other Cancer** |  |  |  |  |  |  |  |  |
| None | Reference |  | Reference |  | Reference |  | Reference |  |
| Skin Cancer Other than Melanoma | NA | NA | 1.54 (-0.53, 3.61) | 0.144 | 1.53 (-0.51, 3.57) | 0.142 | 1.86 (-0.18, 3.91) | 0.074 |
| Other Cancer Excluding Non-Melanoma Skin Cancers | NA | NA | 3.69 (2.31, 5.07) | <0.001 | 3.43 (2.04, 4.81) | <0.001 | 3.77 (2.36, 5.18) | <0.001 |
| **Prior Recurrence Status** |  |  |  |  |  |  |  |  |
| Disease Free | Reference |  | Reference |  | Reference |  | Reference |  |
| Recurrence | NA | NA | 3.56 (1.12, 6.01) | 0.004 | 3.36 (0.95, 5.77) | 0.006 | 4.86 (2.19, 7.53) | <0.001 |
| **Received Care at Home** |  |  |  |  |  |  |  |  |
| Did Not Need Care | Reference |  | Reference |  | Reference |  | Reference |  |
| Needed Care and Got It | NA | NA | NA | NA | 1.06 (-0.06, 2.18) | 0.064 | -0.39 (-1.61, 0.83) | 0.531 |
| Needed Care and Did Not Get It | NA | NA | NA | NA | 2.11 (-0.33, 4.55) | 0.089 | -0.16 (-2.70, 2.38) | 0.903 |
| Other | NA | NA | NA | NA | 4.11 (2.62, 5.61) | <0.001 | 1.57 (-0.08, 3.21) | 0.063 |
| **SF-36 Physical Functioning** ^‡^ | NA | NA | NA | NA | NA | NA | -0.21 (-0.69, 0.27) | 0.389 |
| **Stress Score** ^‡^ | NA | NA | NA | NA | NA | NA | 3.09 (2.65, 3.53) | <0.001 |

Abbreviations: Coef., coefficient; CI, confidence interval. NA, not applicable.

^†^ The coefficients and 95% CIs were per 10-year increase.

^‡^ The coefficients and 95% CIs were per 1 standard deviation increase

**Supplemental Table S4. Recurrence Type by Clinically Significant FCR Status and FCR Score Among Survey Respondents**

|  | **Disease free** | **In situ recurrence** | **Local recurrence** | **Regional recurrence** | **Distant recurrence** | ***p*-value** ^†^ |
| --- | --- | --- | --- | --- | --- | --- |
|  | **N = 1,274** | **N = 7** | **N = 17** | **N = 9** | **N = 17** |  |
|  | **No. (%)** | **No. (%)** | **No. (%)** | **No. (%)** | **No. (%)** |  |
| **Clinically Significant FCR** |  |  |  |  |  |  |
| Non-clinical | 1,041 (81.7%) | 4 (57.1%) | 11 (64.7%) | 7 (77.8%) | 9 (52.9%) | 0.005 |
| Clinical | 233 (18.3%) | 3 (42.9%) | 6 (35.3%) | 2 (22.2%) | 8 (47.1%) |  |
| **FCR Score, mean (SD)** | 13.8 (7.5) | 16.6 (9.0) | 18.2 (5.2) | 15.6 (8.4) | 19.6 (10.2) | 0.002 |

Abbreviations: FCR, fear of cancer recurrence; No., number; SD, standard deviation.

^†^ *P*-value was computed using the *t*-tests or Pearson’s Chi-squared test.

**Supplemental Table S5. Demographic and Clinical Characteristics of Survey Responders and Non-Responders**

| **Characteristic** | **Non-Responders** | **Responders** |
| --- | --- | --- |
|  | N = 1,595 | N = 1,390 |
| **Age, mean (SD)**^†^ | 64.1 (12.6) | 63.1 (11.7) |
| **Years from diagnosis to survey, mean (SD)**^‡^ | 9.4 (5.4) | 9.1 (6.0) |
| **Sex** |  |  |
| Male | 8 (0.6%) | 6 (0.4%) |
| Female | 1436 (99.4%) | 1384 (99.6%) |
| **Race/Ethnicity** |  |  |
| Non-Hispanic White | 923 (58.1%) | 968 (69.6%) |
| Non-Hispanic Black | 547 (34.4%) | 309 (22.2%) |
| Other | 118 (7.4%) | 113 (8.1%) |
| **Marital Status** |  |  |
| Married or Living with a Partner | 817 (60.9%) | 926 (67.3%) |
| Widowed | 75 (5.6%) | 108 (7.9%) |
| Divorced | 122 (9.1%) | 141 (10.3%) |
| Separated | 312 (23.3%) | 24 (1.7%) |
| Single or never Married | 15 (1.1%) | 176 (12.8%) |
| **Insurance** |  |  |
| Not Insured | 5 (0.4%) | 2 (0.2%) |
| Private Insurance | 910 (63.9%) | 945 (72.7%) |
| Medicaid | 101 (7.1%) | 62 (4.8%) |
| Medicare | 383 (26.9%) | 269 (20.7%) |
| Other Insurance | 24 (1.7%) | 21 (1.6%) |
| **Alcohol Consumption** |  |  |
| Non-drinker | 586 (45.1%) | 601 (44.1%) |
| Previous Drinker | 43 (3.3%) | 188 (13.8%) |
| Current Drinker | 671 (51.6%) | 575 (42.2%) |
| **Smoking** |  |  |
| Non-smoker | 817 (61.8%) | 891 (65.1%) |
| Previous Smoker | 138 (10.4%) | 399 (29.1%) |
| Current Smoker | 366 (27.7%) | 79 (5.8%) |
| **Tumor Stage** |  |  |
| 0 | 268 (18.8%) | 238 (17.9%) |
| I | 591 (41.5%) | 622 (46.8%) |
| II | 432 (30.4%) | 342 (25.7%) |
| III | 132 (9.3%) | 127 (9.6%) |
| **Tumor Grade** |  |  |
| 1 | 189 (13.8%) | 177 (14.1%) |
| 2 | 651 (47.7%) | 559 (44.5%) |
| 3 | 525 (38.5%) | 518 (41.3%) |
| 4 | 0 (0.0%) | 1 (0.1%) |
| **Estrogen Receptor** |  |  |
| Neg | 277 (20.5%) | 269 (21.6%) |
| Pos | 1071 (79.5%) | 975 (78.4%) |
| **Progesterone Receptor** |  |  |
| Neg | 417 (31.1%) | 413 (33.3%) |
| Pos | 924 (68.9%) | 828 (66.7%) |
| **HER2 Status** |  |  |
| Neg | 934 (83.5%) | 857 (83.1%) |
| Pos | 185 (16.5%) | 174 (16.9%) |
| **Tumor Subtype** |  |  |
| HR+/HER2- | 842 (66.9%) | 690 (67.0%) |
| HR+/HER2+ | 145 (11.5%) | 113 (11.0%) |
| HR-/HER2+ | 69 (5.5%) | 61 (5.9%) |
| TNBC | 202 (16.1%) | 166 (16.1%) |
| **Had Surgery** |  |  |
| None | 12 (0.9%) | 8 (0.6%) |
| Lumpectomy | 873 (61.9%) | 694 (52.2%) |
| Mastectomy | 402 (28.5%) | 470 (35.4%) |
| Bilateral Mastectomy | 124 (8.8%) | 157 (11.8%) |
| **Chemotherapy** |  |  |
| No | 832 (57.6%) | 738 (55.2%) |
| Yes | 612 (42.4%) | 598 (44.8%) |
| **Radiotherapy** |  |  |
| No | 565 (39.2%) | 508 (38.1%) |
| Yes | 875 (60.8%) | 824 (61.9%) |
| **Hormone Therapy** |  |  |
| No | 466 (32.3%) | 447 (33.5%) |
| Yes | 978 (67.7%) | 889 (66.5%) |
| **Other Cancer at Diagnosis** |  |  |
| No | 1402 (97.1%) | 1283 (97.0%) |
| Yes | 42 (2.9%) | 40 (3.0%) |
| **Prior Recurrence Status** |  |  |
| Disease Free | 1309 (93.0%) | 1274 (96.2%) |
| Recurrence | 98 (7.0%) | 50 (3.8%) |
| **Comorbidity Index** |  |  |
| 0 | 1237 (85.7%) | 1136 (86.2%) |
| 1 | 157 (10.9%) | 87 (6.6%) |
| 2+ | 50 (3.5%) | 95 (7.2%) |

^†^ For non-responders, age was calculated as the interval between the date of birth and the survey distribution date.

^‡^ For non-responders, the time from diagnosis to survey was calculated as the interval between the date of diagnosis and the survey distribution date.
